# Fractional Calculus Model of Electrical Impedance Applied to Human Skin

**DOI:** 10.1371/journal.pone.0059483

**Published:** 2013-04-05

**Authors:** Zoran B. Vosika, Goran M. Lazovic, Gradimir N. Misevic, Jovana B. Simic-Krstic

**Affiliations:** 1 Department of Biomedical Engineering, Faculty of Mechanical Engineering at University of Belgrade, Belgrade, Serbia; 2 Department of Mathematics, Faculty of Mechanical Engineering at University of Belgrade, Belgrade, Serbia; 3 Department of Research, Gimmune GmbH, Zug, Switzerland; University of California at Berkeley, United States of America

## Abstract

Fractional calculus is a mathematical approach dealing with derivatives and integrals of arbitrary and complex orders. Therefore, it adds a new dimension to understand and describe basic nature and behavior of complex systems in an improved way. Here we use the fractional calculus for modeling electrical properties of biological systems. We derived a new class of generalized models for electrical impedance and applied them to human skin by experimental data fitting. The primary model introduces new generalizations of: 1) Weyl fractional derivative operator, 2) Cole equation, and 3) Constant Phase Element (*CPE*). These generalizations were described by the novel equation which presented parameter 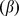 related to remnant memory and corrected four essential parameters 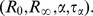 We further generalized single generalized element by introducing specific partial sum of Maclaurin series determined by parameters 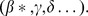 We defined individual primary model elements and their serial combination models by the appropriate equations and electrical schemes. Cole equation is a special case of our generalized class of models for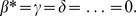 Previous bioimpedance data analyses of living systems using basic Cole and serial Cole models show significant imprecisions. Our new class of models considerably improves the quality of fitting, evaluated by mean square errors, for bioimpedance data obtained from human skin. Our models with new parameters presented in specific partial sum of Maclaurin series also extend representation, understanding and description of complex systems electrical properties in terms of remnant memory effects.

## Introduction

Application of the mathematics in biology and medicine requires interdisciplinary approach employing the high level of theoretical and experimentally based knowledge in all three disciplines [1],[2]. Here we will briefly introduce thematically relevant overview of the previous use of fractional calculus related to electrical impedance with application to human skin. Fractional calculus is a branch of mathematical analysis that generalizes the derivative and integral of a function to non-integer order [3],[4]. Application of fractional calculus in classical and modern physics greatly contributed to the analysis and our understanding of physico-chemical and bio-physical complex systems [5]. In the past two decades fractional calculus extended popularity in other natural science branches such as chemistry, biology and medicine. Living organisms are the most complex systems composed of over billons of different interconnecting entities at different spatial and temporal scales [6]. Therefore, our understanding of biological systems organization requires fractional calculus as a mathematical tool [5],[7],[8],[9],[10]. A large number of useful biophysical studies reported applications of fractional calculus; however, they were limited to relatively small number of biological model system examples such as: (1) electrical properties of neurons in neurobiology [11], (2) viscoelastic properties of muscles and bones in tissue bioengineering [12], [13], (3) kinetic properties of cell growth and differentiation during morphogenesis in developmental biology [14].

Fractional calculus is a mathematical field extending classical calculus for non-integer order of derivation thus dealing with derivatives and integrals of arbitrary and complex orders [3]–[5], [8], [15]. The fractional derivatives are non-local operators because they are defined using integrals. Consequently the fractional derivative in time contains information about the function at earlier points, thus it possesses a memory effect, and it includes non-local spatial effects [3]–[5], [8], [15]. In other words fractional derivatives are not a local property (point – quantity) and they consider the history and non-local distributed effects which are essential for better and more precise description and understanding of the complex and dynamic system behavior.

Fractional calculus applications in life sciences provides possibility to analytically focus on modeling of biological life processes where fractional order model will span multiple scales (nanoscale, microscale, mesoscale, and macroscale). Skin is the largest human organ with extremely high cellular and molecular complexity functioning as the protecting, communication and transfer interface between body and environment [16]. Therefore, human skin as highly ordered multilayer organ is particularly suitable model system for applying fractional calculus approach. Commonly, structural and functional studies of human skin employed measurements of bioelectrical and biochemical properties as well as simplified modeling. These approaches were incapable to provide mathematically precise analytical information and statistically significant predications on the electrical behavior of skin. Here we are extending fractional calculus application in biomedical field of natural sciences by modeling electrical properties of human skin which are based on its structural components and thus of its physiological state. Conductance and dielectric features of material, including biological tissues, are known to exhibit frequency dispersions [17], [18]. Impedance is therefore a complex resistivity (real and imaginary part) displayed under alternative current. We have used fractional calculus to model electrical impedance and applied derived models to describe bioimpedance properties of human skin as a test system.

Multifrequency measurements and modeling of electrical impedance is an important spectroscopy method in study of complex biological tissues and materials such as human skin. Passive electric properties of human skin were studied by Cole mathematical method employing bioimpedance measurements below 100 kHz [17], [19]. Cole model deals with both conductive and dielectric properties [20], whereas Cole-Cole approach primarily describes dielectric features (determined as permittivity) [21]. Since the human skin, as the complex organ, displays both conductive and dielectric behavior, neither of the two models can be applied to precisely describe and study bioimpedance properties of this organ.

Biological membranes show a high capacitance and a low but complicated pattern of conductivity [20]. Biological tissues as complex multi-layer systems behave as an anisotropic material due to the variable orientation of cells and their plasma membranes. As mentioned above in 1940 Cole formulated a mathematical model of electrical properties of cell membranes based on impedance measurements at multi frequency alternative current. Kenneth Cole and Robert Cole have conveyed another mathematical model of dielectric properties for materials in 1941 [21]. In 2001 El-Lakkani [22] attempted to analyze electric and dielectric properties of different types of human tissues either by Cole-Cole model [20] or Dissado model [23] in the alternative current frequency range from 20 Hz to100 kHz. This type of modeling was reviewed by Grimnes & Martinsen [17]. Such Multi Frequency Bioelectrical Impedance Analysis (MF-BIA) is a noninvasive and relatively new technique for studying biological systems. The complex impedance as a function of frequency of the external alternating voltage source (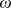 is frequency 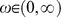) is one of the powerful linear passive characteristic of materials in the frequency domain. One of the passive characteristics of materials in alternating current circuits models is a Constant Phase Element (*CPE*) which will be here further mathematically defined and generalized by impedance equations using fractional calculus approach.

We have used fractional calculus approach to construct simple models with unified principles. Without fractional calculus approach it would not be possible to make this generalized type of superior and more precise class of models where Cole model is a special case. Here we report modeling of bioimpedance using fractional calculus approach and experimental data fitting for human skin test system. We have derived new bioimpedance equations introducing one new parameter and corrections for four parameters by employing generalized Weyl fractional derivative operator. Our model and results provide significant mathematical advance for solving complex biosystems when compared to the classical Cole model. Therefore, presented bioimpedance fractional calculus modeling may be useful for further fundamental research with applications in medicine which are related to physiological and pathological analysis of human skin.

## Results

Our modeling strategy of the bioimpedance with application to human skin is based on generalization of Weyl fractional derivative operator, generalization of Cole equation, and generalization of *CPE*.

### 1. Generalized Weyl fractional calculus

Our primary idea was to generalize Weyl fractional calculus because this method is necessary for mathematical analysis of complex periodic functions describing characteristic values of alternating current in electric circuits which are employed in bioimpedance modeling.

#### 1.1. Introduction to Weyl fractional calculus

In 1917 Weyl introduced his new approach of fractional calculus to analysis of periodic functions. In summary the 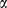 -th fractional integral and 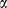 -th fractional derivative of a 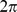 periodic adequate complex function of real variable are defined respectively by
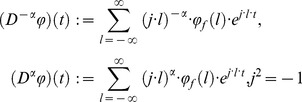
(1)where 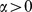 and



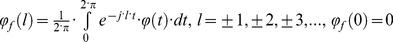
(2)This discrete Fourier transform can be viewed as the most usual way of defining fractional integrals and derivatives of periodic functions. The Equations (1) are correctly defined under the condition that 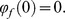 Supplement to definition (1) is 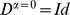 where 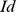 is unit operator. In the case of considering formula for Weyl fractional integral
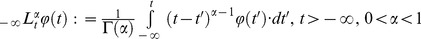
(3)for
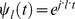
(4)then it follows (Butzer and Nessel, [9])
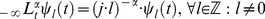
(5)hence, if we define
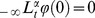
(6)then it follows



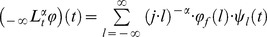
(7)This operator 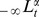 is identical to the operator 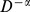 for 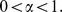 Then the Equation (7) can be written in the following form
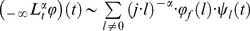
(8)


Previously defined operator is linear
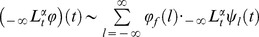
(9)


#### 1.2. Rigorous treatment of the Weyl approach to fractional calculus

The Weyl approach to fractional calculus, can be rigorously treated in the Banach spaces 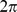 periodic complex function of real variable 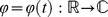, defined by the interval 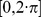


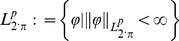
(10)with norms of this function defined by



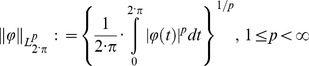
(11)The factor 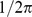 is characteristic for the usual Fouirer analysis. In the case 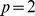 describes Hilbert space 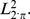 Dot product and norm are defined in the standard way (over line means complex conjugation).
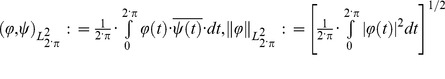
(12)


If 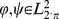 and 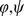 are real functions the following relation holds
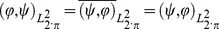
(13)


As described by Butzer and Westphal [24], Equation (8) can be considered as a motive for the definition of fractional integral 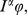 in the form of the convolution integral for 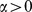 and 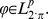 In order to more accurately describe the fractional integral of Weyl 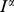 for 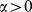 and 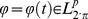 the right side of Equation (8) will be rewritten as
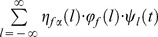
(14)with



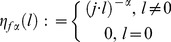
(15)The corresponding Fourier transformed function is
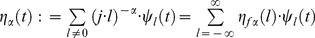
(16)


We now move to more precise definition of Weyl fractional integral. This function will be used as a kernel of the associated convolution integral, so-called Weyl fractional integral 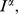 defined as follows
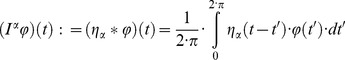
(17)


Then according to Butzer and Nessel, [9], 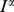 bounded linear operator, is actually a continuous operator over the space 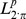 for all 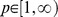


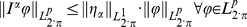
(18)


Moreover, the convolutional theorems for periodic functions (Butzer and Westphal, [24], Theorem 4.1.3), will be
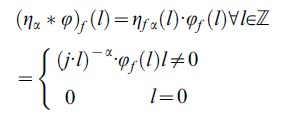
(19)


Comparing Equations (1) and (8) for the linear operator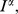 it can be reasonably concluded that 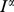 is a fractional integral operator. According to the Equation (17)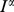 is defined
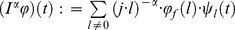
(20)


Since 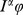 defines for all 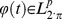 then 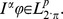 Same relation does not hold for Weyl fractional derivative
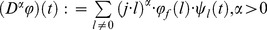
(21)


If the index 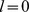 then the corresponding member in Equation (21) is equal zero. For 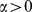 the function 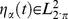 because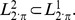 In the particular case when 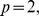 for the Hilbert space 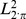 Parseval Equation gives the maximum definitional domain for fractional derivative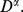 This domain is defined by
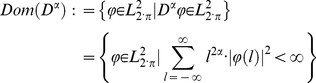
(22)


To eliminate the obvious deficiency, one of the ways is to define the operator 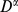 in the theory of distribution. Although we will not consider such general problem, we will use the most appropriate fractional operators and his domains. Later we will derive generalizations of fractional operators and their domains. Furthermore, we will consider the existence of a nontrivial, nonempty, common domain for all fractional integrals 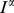 and derivatives 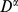 for 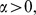 which will be also codomain.

In this regard, we will review the maximal set 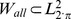 of complex functions of a real variable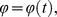 so that following is valid.
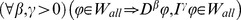
(23)


It follows that the set of 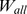 will define Weyl fractional derivatives and fractional integrals, so that the 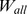 is domain and codomain. Previously mentioned nonempty set 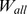 (e.g., function 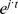 belongs to such a set) is by construction closed under the operation of Weyl fractional integrals. The idea for the basic theorem of generalized Weyl fractionation derivatives and integrals is contained in the following theorem.

#### Theorem 1

For all 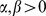

*(i)* 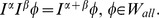

*(ii)* 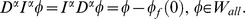

*Proof:* We should bear in mind the sum member 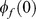 must be excluded. The proof is analogous to that given in (Butzer and Westphal, [24], Proposition 4.1).

#### 1.3. Generalization of Weyl fractional integral and derivative

Previous mathematical description is the basis for generalization of Weyl fractional integral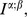 for 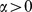 and 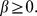 Motivation for introducing generalization of Weyl fractional integral 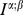 is to use useful modification of Riemann –Liouville fractional integral 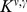 described by Nigmatullin [15]. Our basic idea for a new type of generalization is to use fractional integral operator acting on a periodic function so that the result of the operator action is a periodic function. For that purpose the set of Equations (14), (15) and (16) will be written in more general form in account member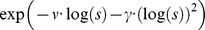 from Nigmatullin`s Equation [15].
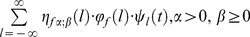
(24)and
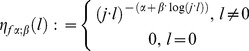
(25)respectively,
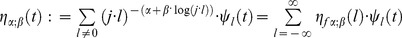
(26)


#### Lemma 1


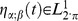
 for all 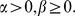


*Proof* For 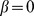


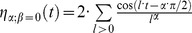
(27)which is a convergent series in 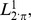 if 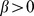 then



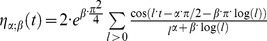
(28)This series is also convergent with respect to the previous, because there is a functional dependence of the exponent denominator fraction by the member of the sum.

#### Theorem 2

Generalization of Weyl fractional integral 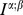


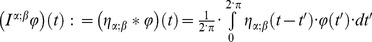
(29)represents a linear and bounded, actually continuous operator in 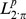 for all 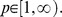


*Proof:* The operator 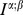 is bounded because the following is valid.
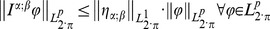
(30)


Norm of the first element to the right is finite because of the previous lemma.

Therefore, if 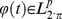 then 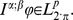 As with the Weyl fractional derivatives, there is a problem of completeness of the domain operator if other real value for 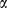 (non zero) and 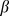 are used. In general case instead of 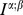 we use 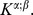 Therefore, we define the operator 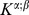 acting on the functions 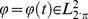 which are written
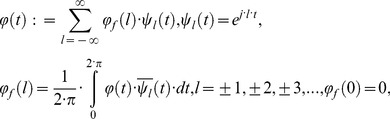
(31)


In the case that the operator 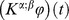 makes sense, analogous to (1) we define:
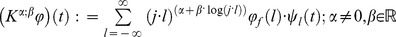
(32)


Supplement to the previous definition is 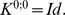


The idea presented in the previous section on non-trivial and non-empty common domain and codomain for all operators that are defined by formula (32) will be used here. By analogy we define domain of the operator 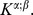


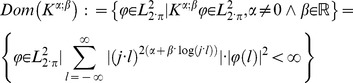
(33)


This domain is neither equal to 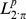 nor to 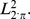 As indicated previously here we will define maximal set 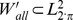 of complex functions of a real variable 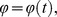 with set properties.



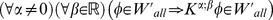
(34)


Described non-empty set 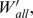 which is domain and codomain for all described operators 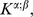 is by construction closed in relation to the generalization of Weyl fractional derivative or integral.

#### Theorem 3

The basic properties of operators in a given domain 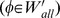




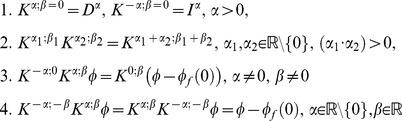
(35)


*Proof:* For 1–3 is obvious and for 4 it can be considered if 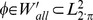 then this function can be written in a unique way 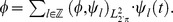


Because 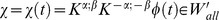 for 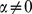 it follows
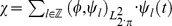
then for all 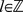




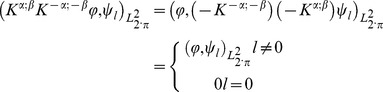
therefore



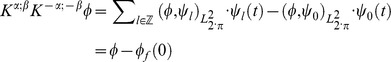



In a similar way proofs are provided for the other three equations.

Also, instead 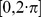 it is possible to use a symmetrical segment 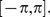 In the case the periodic function 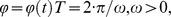 appropriate formulas will be done by the transformation 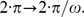 Then instead 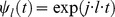 we write 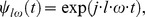 etc. In this case, if operator 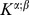 acts on 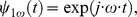 then following holds
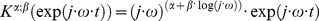
(36)


The previous operator is analogous to the following one
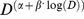
(37)in the case when the Weyl operator derivatives is true 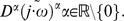


If one introduces a non-negative relaxation time parameter 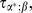 the modified operator 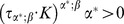 for operator of fractional type derivative) acts as follows
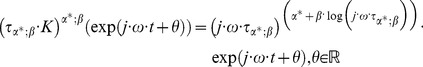
(38)


If 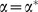 and 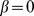 then operator 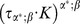 is fractional derivative of Weyl type
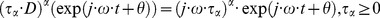
(39)


Using appropriate mathematical operations and weakened conditions described in Theorem 3 the Equation (38) can be reduced to form (36) due to specific functional dependence. Subsequently we will use operators 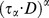 and 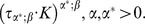


### 2. Generalized Cole element and Constant Phase Element *(CPE)*

Multi Frequency Bioelectrical Impedance Analysis (MF-BIA) is a noninvasive technique for studying biological systems. The complex impedance as a function of frequency of the external alternating voltage source (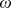 is frequency, 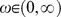) is one of the linear passive characteristic of materials in the frequency domain. In alternating current circuits the Constant Phase Element of capacitance type such as 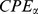 ([7], [8], [25]) is defined by the impedance equation
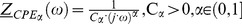
(40)

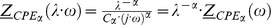




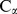
 is capacitance of order 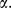 For fractional index 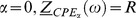 (resistance).

**Figure 1:**
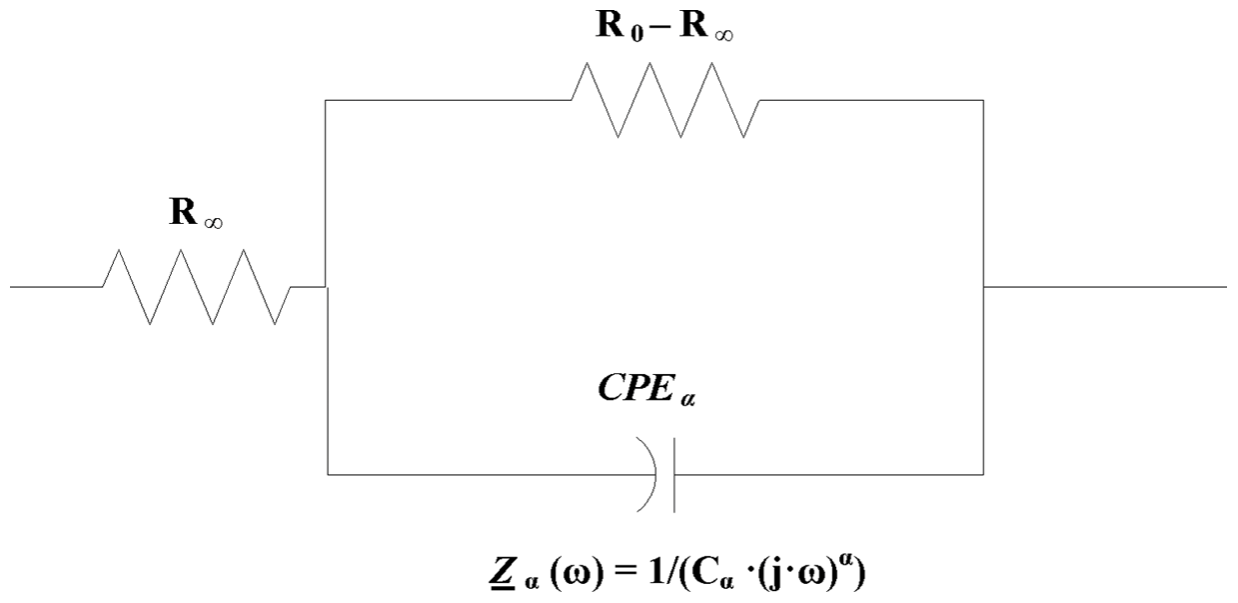
Schema of circuit for modified Cole element [20] according to Magin [7], [8].

Mathematical model of electrical properties of cell membranes based on impedance measurements at multi frequency alternative current was firstly formulated by Cole in 1940 [20]. Magin [7], [8] derived and generalized Cole equation using fractional calculus. His model comprises of three hypothetical circuit elements: a low-frequency resistor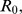 a high-frequency resistor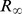 and 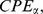 arranged as shown in Figure 1. This circuit can be also visualized as two serial connected elements, where the first one is 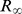 and second one is 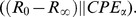 Here we name the second element as reduced Cole type element.

The complex impedance described by Cole model is given by the following equation [20].
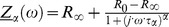
(41)


Here is the characteristic relaxation time according to Magin [7],[8], positive constant.
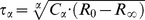
(42)


All of the four following parameters 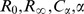 are material constants independent of frequency.

Here we derive Cole equation (41) for the circuit shown in Figure 1. Applied alternating voltage to the system is 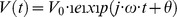 (*V_0_* is the voltage amplitude, θ is the phase angle between the voltage and the current), current passing through the system is 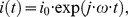 and strength of current amplitude is *i_0_*. Impedance of the system is given by the equation
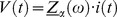
(43)Where the relations describing the system 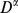 (the Weyl fractional derivative,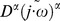) is



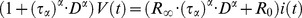
(44)In terms of fractional derivatives usage, our Eq. (2.44) has some analogy to the modified Zener model of a viscoelastic body [26] and Bohannan equation [27]. However, Bohannan applied fractional derivative according to Riemann-Liouville, whereas we have done according to Weyl. The second difference is that Bohannan [27] is deriving Cole-Cole equation [21], which is describing frequency dispersion of complex dielectric constant, and we have derived Cole equation [17], [20], which is describing frequency dispersion of impedance. If one takes into account geometrical properties of the physical system, such as surface of electrode and distance between them, complex dielectric constant becomes complex capacitance (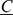). Note that in the theory of alternative current the admittance 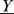 is described by formula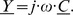 The Eq. (2.44) describes frequency dispersion of complex resistivity in the case when geometry is not taken into account.

In order to generalize our previous Eq. (2.44) we have used similar principal of mathematical approach as reported by Nigmatullin [15]. They described that for a strongly correlated fractal medium a generalization of the Riemann–Liouville fractional integral is obtained. In this paper we have modified generalization of Riemann-Liouville fractional integral and derivative for the case of periodic functions and obtained a new type of generalization of Weyl fractional integral and derivative.

Formally in Equation (44) replacement is done by
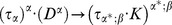
(45)


By introducing new parameters for resistance 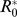 and 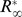 in Equation (44) we derive next equation
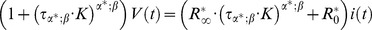
(46)Then in (43) we perform following change 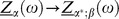 to obtain



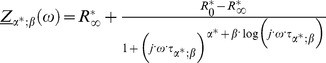
(47)The Equation (47) describes our new generalized Cole model based on fractional calculus (our primary model). Five new physical and phenomenological parameters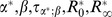 were introduced. In our work we have introduced new 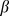 parameter for modeling electrical impedance of complex systems. This parameter has formal mathematical analogy to 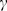 parameter presented by Nigmatullin [15]. Parameter 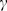 describes relaxation properties of dielectric phenomena of the medium and is derived as the generalized equation of the well-known Kohlrausch Williams Watts relaxation law. Constants 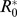 and 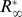 are the corresponding resistances, therefore, we can write.
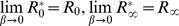
(48)


It should be mentioned that for small 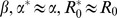
and 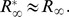 The 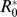 and 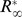 constants have different meaning to those described by the Cole model (Equation (41)), because the leading members of Equation (47) 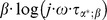 and 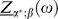 have different asymptotic behavior when 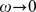 and 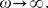


**Figure 2:**
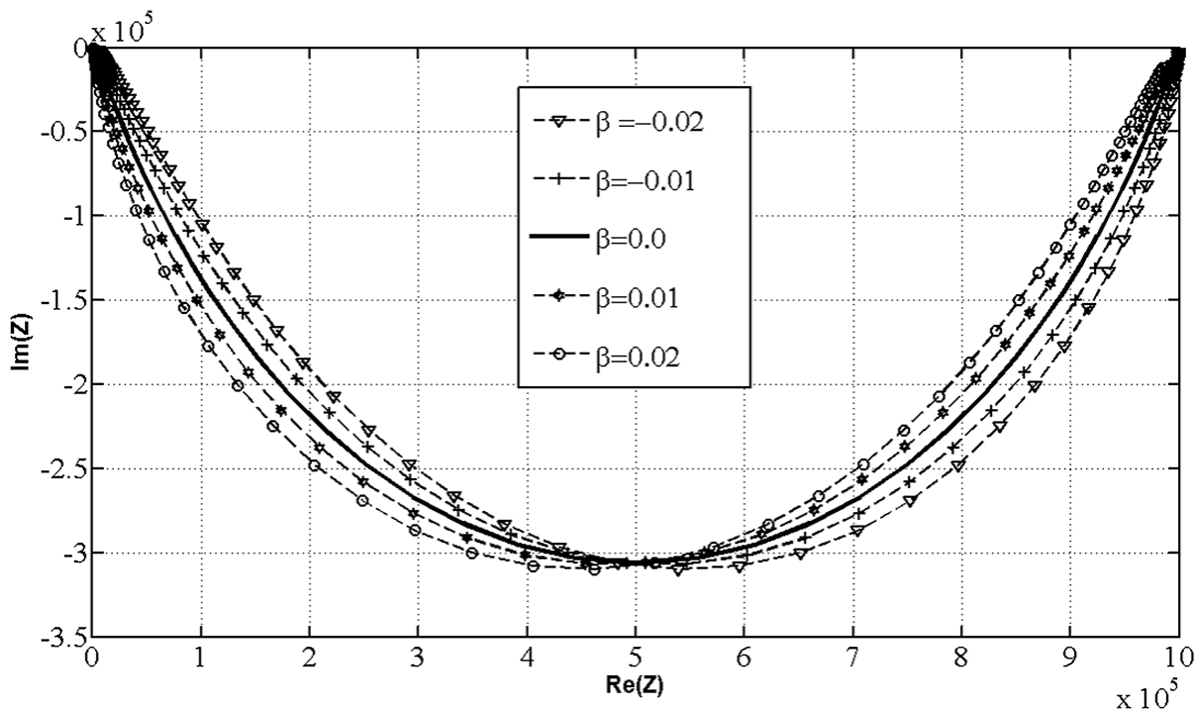
Cole Plot of Generalized Cole Model (GC1). Cole plot of GC1 model with five selected values of β parameter and suitable fixed parameters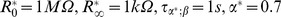 in the frequency interval 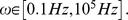

Using our model we have tested the effects of 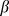 on 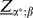 while keeping other parameters constant. The example of Cole plot based on equation (47) showed that for values of 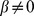 is not circular arc as in Cole model, Figure 2.

If we assume that the change of conducting properties of electrical circuits is only in the *CPE*, then *CPE* can be replaced and we can write.
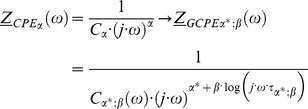
(49)


Cole model uses linear scaling as seen from the above equation. For generalized CPE (GCPE) we are scaling frequency for dispersion of impedance in non-linear manner. This can be seen from equation (49) i.e. scaling with function not only with one constant α value. Therefore, we have non linearized and non-constant scaling with two parameters 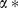 and 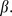 This is important advantages for modeling and describing natural complex systems. It should be noted that in Cole model linear scaling is valid and this is only one special case of our generalized model where 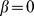 and non-generalized CPE.

The function 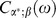 is such a function of frequency where the non-negative constant 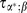 can be described by the following self-consistent equation.
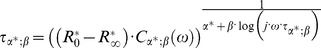
(50)

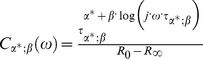



**Figure 3:**
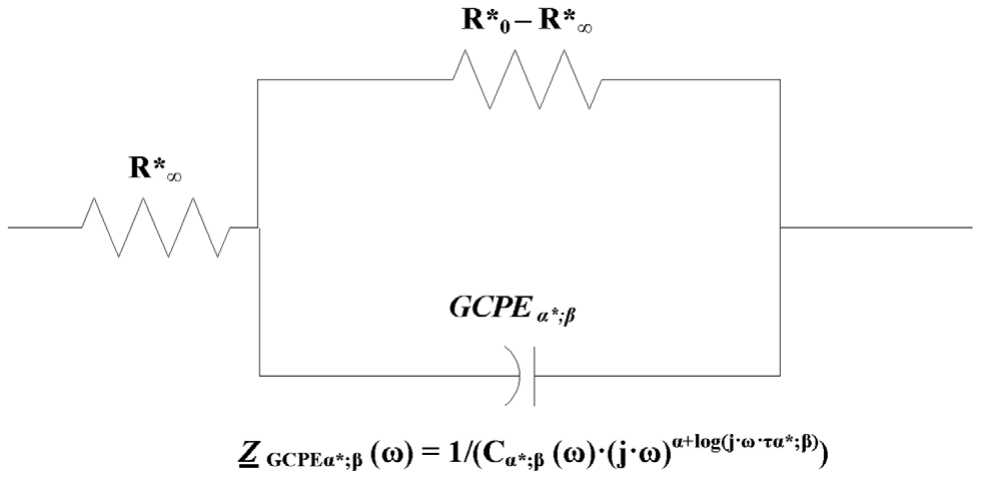
Schema of Circuit for Generalized Cole (GC1) Element with GCPE.

The Equation (49) introduces the element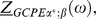 and provides generalization of *CPE* (*GCPE)*. The electrical circuit comprising of the element 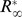 in serial connection with the elements 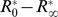 and 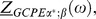 which are themselves in a special parallel connection, is described by the Equation (47) and schematically presented in Figure 3. This means that 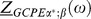 is a function depending on the element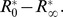 Part of the electric circuit shown in Fig. 3, which includes parallel connection of *GCPE* element with 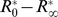 element, we name a reduced generalized Cole element. It should be noted that the following functional dependence of impedance 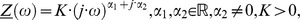 described by Nigmatulin and Mehaute [28], is only the special case of the element 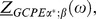 whereas our equation describes the generalized case. Nigmatulin`s equation [15] naturally explains temporal irreversibility phenomena which can appear in linear systems with remnant memory, while our model defines more complex behavior compared to the mentioned power law scenario. For the operator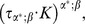 described in the Equation (46), limitation values for 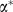 and 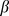 do not have to be strict as in the case for 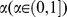. Mathematically formulated our model is introducing a new parameter 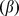 and four corrected parameters 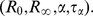


Using fractional calculus approach we have derived a new class of models for electrical impedance by generalizing Weyl fractional derivative operator, Cole model and constant phase element. These generalizations were described by the novel equation which presented parameter 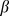 which can adopt positive and negative values. Therefore, for value of parameter 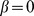 Cole model is a special case of our generalized model. Our generalized equation (47), containing non-integer integrals and derivatives either with real or complex power-law exponents, naturally explains temporal irreversibility phenomena. According to Nigmatullin and Trujillo these partial irreversibility phenomena can be declared as “remnant” memory of the complex system [15]. It would mean that in complex systems containing many entities only one part of their microscopic states will be conserved on the following level of intermediate scales and expressed in the form of the fractional integral.

We have derived a new primary model (equation 47) using fractional calculus approach for generalization of CPE and Cole equation together with introduction of a new parameter 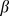 which according to fractional calculus and Nigmatullin and Trujillo [15] we relate and interpret as remnant memory of the system. For values of parameter 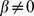 our model defines the system with remnant memory and is represented in an impedance plot not as a circle arc but as a ellipsoid like arc type. For values of 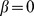 the model does not take in account eventually existing remnant memory. According to the equation (47) our model is then deduced to the special case of Cole model, represented as a circle arc in an impedance plot, describing the system without taking in account remnant memory.

Our new parameter 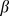 adopting non restricted positive and negative values is introducing remnant memory. This is a new parameter different to 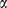 which can adopt only values between 0 and 1and is usually interpreted as a distribution of relaxation times [17], [19], [29].

Different mathematical approaches for studying electrical properties of human skin were attempted using impedance models based on serially connected reduced (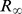 excluded) Cole elements (C1) (Yamamoto & Yamamoto [29]). They can be summarized in the following equation (Barsoukov and Macdonald [30]) for 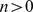


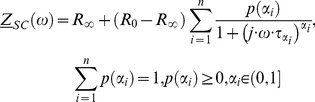
(51)


**Figure 4:**
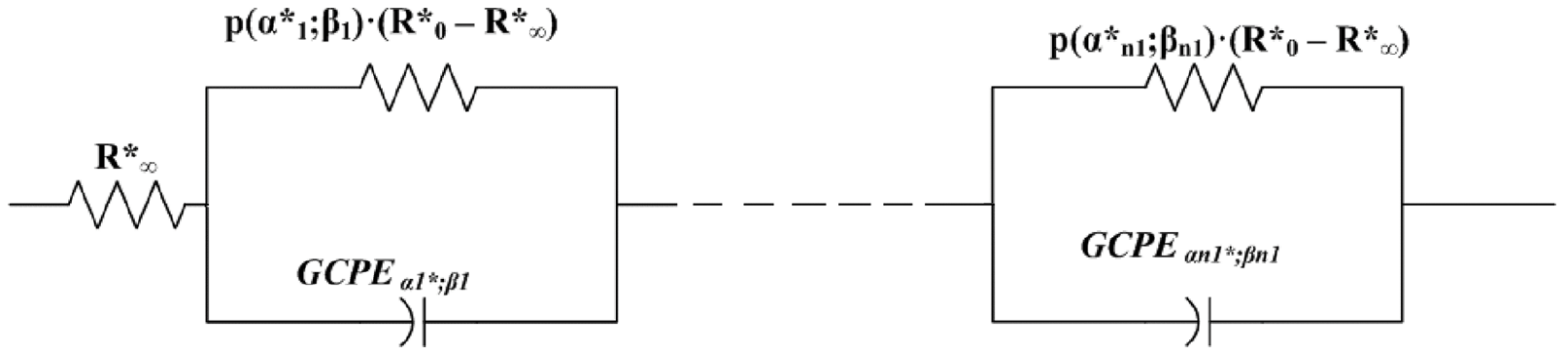
Schema of Circuit for Serially Linked Reduced Generalized Cole Elements.

Here we develop a second model based on serial connection of reduced generalized Cole elements (Equation (52) and electric circuit, Figure 4). This new type of a serial model is based on the generalized bioimpedance Equation (47).
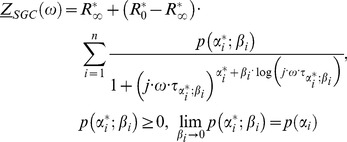
(52)


The Equation (52) is direct generalization of Equation (51).

The Equation (53) denotes our combinatorial bioimpedance model using all permutations of serial connections between reduced Cole elements (Equation (51)) and our reduced generalized Cole elements described in Equations (52).
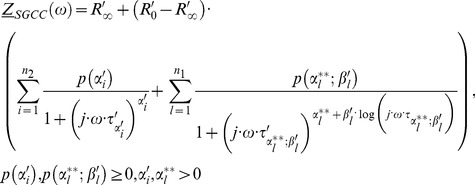
(53)


**Figure 5:**
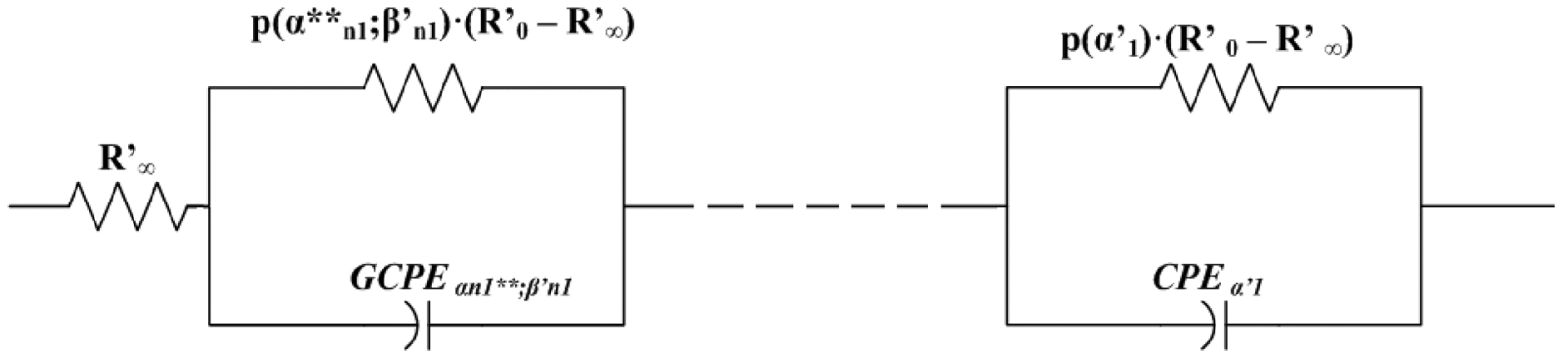
Schema of Circuit for all Permutation of Serially Linked Reduced Generalized Cole Elements and Reduced Cole elements.

A particular permutation case for our third model is using serial connection of reduced Cole elements and reduced generalized Cole element and is based on Equation (53) and electric circuit shown in Figure 5.

For the purpose to set Equation (47) in more general form we have extended previously introduced members 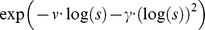 from Nigmatullin's Equation [15] with three more members 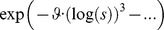


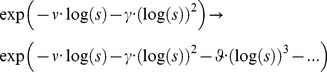
(54)


Formally from the equation (47) it follows
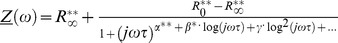
(55)


The equation (55) represents further generalization of our generalized Cole (GGC1) including additional parameters. We have introduced partial sum of Maclaurin series determined by parameters 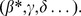 Cole equation is a special case of our generalized class of models for
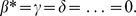


The purpose of this further generalization was to cover the broader frequency dispersion range using one element instead of using serially connected elements with larger number of parameters and inferior fitting.

## Materials and Methods

### 1. Bioimpedance measurements

Bioimpedance skin measurements were performed at University of Belgrade on upper arm of human volunteers with Solartron *1255* Frequency Response Analyzer in combination with Solartron *1286* Pstat/Gstat. Experiments were done in shielded Faraday caged room. The linearity of all measurements with both electrode sizes was confirmed by testing the system with Solartron Schlumberger 12861 test module. The electrodes were made of stainless steel. We have used electrodes with diameter of 0.25 cm and 2.0 cm. The distance between outer edges of two electrodes was 5 cm. The electrode was completely covered with minimal amount of highly conductive cream (3.3 S/m) Grass EC33 obtained from Grass technologies. This cream is specifically designed for skin resistive and conductive measurements. Cream covered electrodes were gently placed on skin in order to avoid putting excess pressure to skin. Total required time for the frequency sweep measurement was about 10 minutes at 22°C and 50% relative humidity, thus, minimalizing artifacts production during measurements due to long cream exposure or cream penetration to skin, as well as sweeting. Error of measurements was <0.1%. Twenty series of measurements were taken at each of the 61 different frequencies ranging between 0.1 Hz and 100.0 KHz. The applied voltage of alternating current was of 1.0 V amplitude. Total required time for the frequency sweep measurement was about 10 minutes.

1. Bioimpedance skin measurements were performed at University of Belgrade,
2. University of Belgrade Review Board (IRB) approved specifically use of oral consent for bioimpedance skin measurements on healthy human volunteers for this study. Please note that bioimpendance skin measurements are noninvasive and have been commonly used in public fitness centers for measurements of body fat content without medical or ethic commission authorizations and are thus generally deemed unnecessary for further ethics commission approval.
3. Each volunteers provided oral consent for bioimpendance skin measurements analyzed and reported in our study.

### 2. Experimental data fitting

Levenberg-Marquardt (LM) nonlinear least squares algorithm L2 (*L_2_*-norm) [31], [32], with Levmar in Octave programming environment was used for fitting experimental data with different models [33]. Without complications this fitting calculation could use maximally ten parameters. This restriction encourages the implementation of LAPACK libraries in *C/C++*.

## Discussion

### 1. Experimental measurements of bioimpedance on human skin

**Figure 6:**
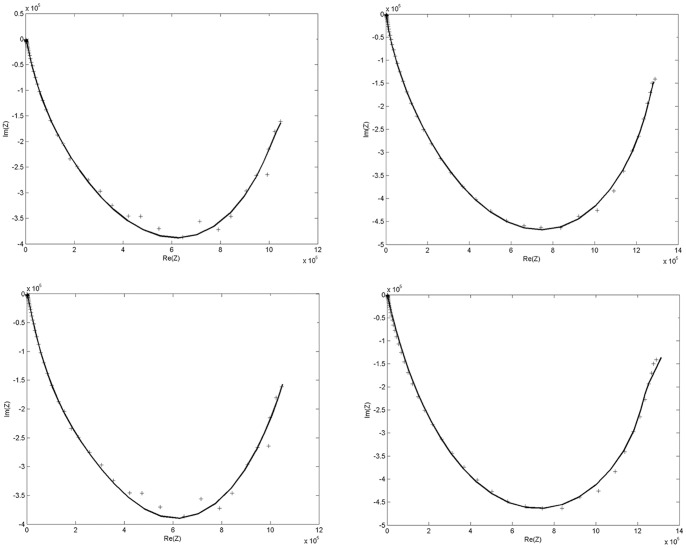
Cole Plot for Experimental Data and LM data Fitting. *Top Left*: Crosses represent experimental data of measurements using electrode *d = 2* cm for *V_0_ = 1.0 V*. Solid line represents LM fit for GGC1 model. *Top Right:* Crosses represent experimental data of measurements using electrode *d = 2.0* cm for *V_0_ = 1.0 V*. Solid line represents LM fit for GGC1 model. *Bottom Left*: Crosses represent experimental data of measurements using electrode *d = 0.25* cm for *V_0_ = 1.0 V*. Solid line represents LM fit for GC2 model. *Bottom Right:* Crosses represent experimental data of measurements using electrode *d = 2.0* cm for *V_0_ = 1.0 V*. Solid line represents LM fit for C1GC1 model.

Bioimpedance measurements on human skin were performed under the conditions described in the experimental methods section. Obtained data are presented in Fig. 6 as Cole-Cole plot. In the *left* part of the Figure 6. we show electrical measurements results acquired using electrodes with 0.25 cm diameter and in the *right* part of Figure 6. results acquired using electrodes with 2.0 cm diameter. For both electrode sizes we have obtained arc type behavior when Z imaginary (*Im (Z)*) was plotted versus Z real (*Re (Z)*). There is a difference in the arc shape for two different electrode sizes. The maximal measured value of *Re (Z)* is greater for larger electrode surface then the maximal value of *Re (Z)* obtained for the smaller electrode surface (Fig. 6). The minimal measured value of *Im (Z)* is smaller for the larger electrode surface then the minimal value of *Im (Z)* obtained for the smaller electrode surface (Fig. 6). Therefore, one can observe that bioimpedance depends from the electrode surface area.

### 2. Experimental data fitting with derived mathematical models

We have used Levenberg-Marquardt (LM) nonlinear least squares algorithm for experimental data fitting with following models: (1) comprised of one Cole element (Equation (41)); (2) based on two serially linked reduced Cole elements (C2) (Equation (51) for n = 2); (3) based on three serially linked reduced Cole elements (C3) (Equation (51) for n = 3); (4) comprised of one generalized Cole element (GC1) (Equation (47)); (5) based on two serially linked reduced generalized Cole elements (GC2) (Equation (52) for n = 2); (6) based on one reduced Cole element serially linked to one reduced generalized Cole element (GC1C1) (Equation (53), n_1_ = 1 and n_2_ = 1, first permutation); (7) based on one reduced generalized Cole element serially linked to one reduced Cole element (C1GC1) (Equation (53), n_1_ = 1 and n_2_ = 1, second permutation). Although all models were analyzed using LM data fitting, we present in Table 1 and 2 only results obtained by three best performing models for human skin as the test system. These models are designated according to the abbreviations of corresponding of electric circuit elements. They are: C2, GC2 (schema shown in Fig. 4 for n = 2), and C1GC1 (schema shown in Fig. 5 for n_1_ = 1 and n_2_ = 1). Future experimental electrical biompedance measurements will demonstrate whether the presented seven model types could provide specific description for different organs, tissues and materials.

**Table 1:** LM Fitted Parameters for Impedance Models C2, GC2, C1GC1.

1.0 V, d = 0.25 cm				1.0 V, d = 2.0 cm			
Parameters C2		Parameters GC2		Parameters C2		Parameters C1GC1	
R_0_ (MΩ)	1.120	R^*^_0_ (MΩ)	1.119	R_0_ (MΩ)	1.353	R'_0_ (MΩ)	1.354
R_∞_ (kΩ)	1.911	R^*^_∞_ (kΩ)	0.680	R_∞_ (kΩ)	1.718	R'_∞_ (kΩ)	2.963
**α_1_**	0.743	α^*^_1_	0.819	**α_1_**	0.784	α^**^_1_	0.532
**τ_1_ (s)**	0.266	τ*_1_ (s)	0.240	**τ_1_ (s)**	0.110	τ**_1_ (s)	1.900
**p(α_1_)**	0.267	p(α^*^_1_, β_1_)	0.422	**p(α_1_)**	0.200	p(α^**^_1_, β'_1_)	1.596
**α_2_**	0.851	α*_2_	0.805	**α_2_**	0.831	**α_2_**	0.961
**τ_2_ (s)**	1.414	τ*_2_ (s)	2.883	**τ_2_ (s)**	0.687	**τ_2_ (s)**	2.704
**p(α_2_)**	0.733	p(α*_2_, β_2_)	0.655	**p(α_2_)**	0.800	**p(α_2_)**	0.356
**β_1_**	0.0	**β_1_**	−0.015	**β_1_**	0.0	**β'_1_**	0.024
**β_2_**	0.0	**β_2_**	0.074	**β_2_**	0.0	**β'_2_**	0.0
**Mean square errors (•10^7^)**							
4.19		3.90		3.64		2.76	

**Table 2:** LM Fitted Parameters for Impedance Models GC2, C1GC1, GGC1.

1.0 V, d = 0.25 cm				1.0 V, d = 2.0 cm			
Parameters GC2		Parameters GGC1		Parameters C1GC1		Parameters GGC1	
R^*^_0_ (MΩ)	1.119	R^**^_0_ (MΩ)	0.642	R'_0_ (MΩ)	1.354	R^**^_0_ (MΩ)	1.461
R^*^_∞_ (kΩ)	0.680	R^**^_∞_ (kΩ)	1.060	R'_∞_ (kΩ)	2.963	R^**^_∞_ (kΩ)	2.530
α^*^_1_	0.819	α^**^	0.940	α^**^_1_	0.532	α^**^	0.707
τ*_1_ (s)	0.240	τ* (s)	0.281	τ**_1_ (s)	1.900	τ* (s)	0.604
p(α^*^_1_, β_1_)	0.422	β*	−0.043233	p(α^**^_1_, β'_1_)	1.596	β*	0.010132
α*_2_	0.805	**γ**	−0.131216	**α_2_**	0.961	**γ**	−0.004308
τ*_2_ (s)	2.883	**δ**	−0.054793	**τ_2_ (s)**	2.704	**δ**	−0.007025
p(α*_2_, β_2_)	0.655	**ε**	−0.009557	**p(α_2_)**	0.356	**ε**	−0.002536
**β_1_**	−0.015	**ζ**	−0.000652	**β'_1_**	0.024	**ζ**	−0.000206
**β_2_**	0.074						
**Mean square errors (•10^7^)**							
3.90		3.90		2.76		0.94	

**Figure 7:**
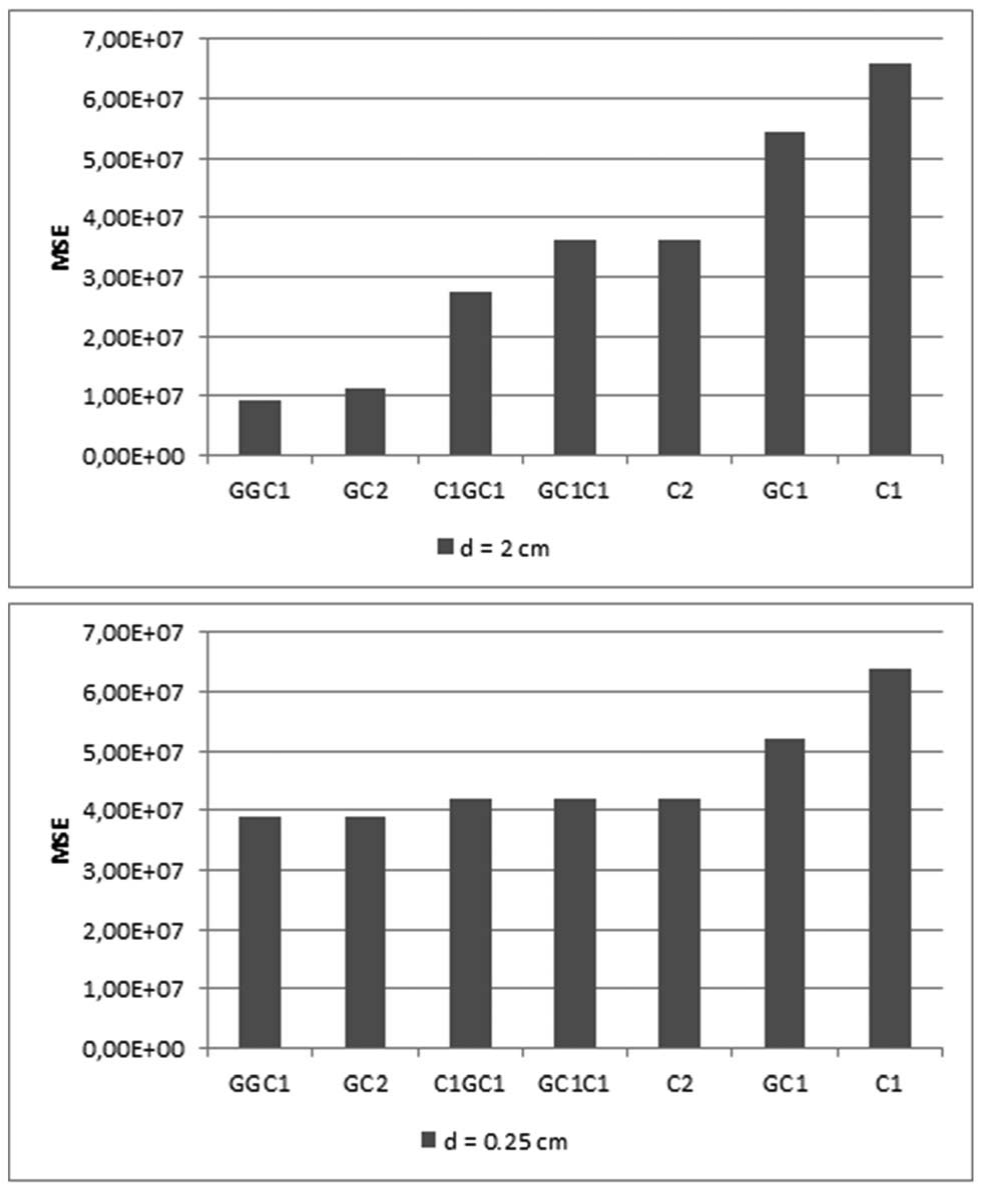
Comparison of model quality evaluated by mean square error. Bioimpedance data fitting with all of our models, either single elements or their serial combination, by Levenberg-Marquardt (LM) nonlinear least squares algorithm for electrode size d = 0.25 and d = 2 cm.

We have started with LM fitting of experimental data using Cole model, GC1, C2 and C3 models in order to obtain initial values for parameter: 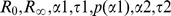 and 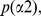 Table 1 and 2. The initial value of 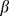 for GC1 was 0.01. Among GC1, C2 and C3 models the C2 model provided the best fitting results (Table 1 and 2). In the second step, we have used the initial values of parameters obtained with C2 model in order to proceed with LM fitting of experimental data using GC2 and C1GC1 and GC1C1 models. The initial values for the parameters 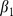 and 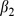 were 0.01. The best fitting results were obtained for GGC1 followed by GC2 and C1GC1 models using the parameter values given in Table 1 and 2 and Figure 7. Bioimpedance data measurements, obtained with two different electrode sizes (0.25 cm and 2 cm in diameter), and fitting data, using GC2 and C1GC1 models, are shown in Fig. 6 and 7. The GC2 model provided the best fitting results for measurements with large electrodes, whereas GC2 and C1GC1 model provided the best fit for measurements with small electrodes, Figure 6. and 7. We have calculated mean squared errors for Cole model and our generalized models derived by fractional calculus. The results showed that our GGC1 model have 85% better experimental impedance data fitting obtained with electrodes of 2 cm diameter and 40% for 0.25 cm electrode diameter Figure 6 and 7. Model types are sorted in the qualitatively same ascending way according to increasing mean square error for both electrode size (d = 0.25 and d = 2 cm) (Fig. 7). These results showed that quality of model is invariant of electrode diameter.

Additional parameters introduced in GGC1 are correction of parameter β improving modeling of remnant memory. Using this particular model type we have further improved bioimpedance data fitting by Levenberg-Marquardt (LM) nonlinear least squares algorithm because this model gave best mean square error values as shown in Figure 7 and Table 1 and 2. In summary our fractional calculus approach adds to better understanding and description of the complex system and their electrical behavior. Therefore, we have presented quantitative evidence about improved precision of our models for description of human skin bioimpedance.

Three serially linked reduced Cole elements had two order of magnitudes higher mean square error and thus is the most inferior quality model.

Taking also in account the Equation (50), it can be concluded that 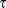 is a function of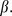 Therefore, it seems that the parameter 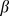 in our impedance model is related to relaxation phenomena of electrical behavior of complex system such as human skin. Since fractional calculus is a mathematical approach dealing with derivatives and integrals of arbitrary and complex orders it adds a new dimension to understand and describe basic nature and behavior of complex processes, such as electrical properties of biological tissues, in an improved way. More precisely it contains in time information about the function at earlier points, thus it possesses a memory effect, and includes non-local spatial effects. Without the use of fractional calculus approach it would not be possible to make our new generalized type of superior and more precise class of models where Cole is a special case.

We have introduced fraction calculus generalization approach in order to be able to cope with complex multi-layered systems with unknown structures which also include simpler structures such as necessary gels and electrodes as additional element influencing and complicating interpretation and analyses of the experimental data.

Our GGC1 (one element) and GC2 (two elements) models provide significantly better fitting of the experimental data then Cole model which is actually, as previously explained, a special case for 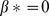 and 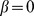 respectively. We have used human skin as one of the examples for complex system and our model is not limited only to such biological material, rather it is a generalized for any complex system including even mixture of biological and not biological systems such as gels and electrodes.

Our generalized *CPE*, generalized Cole model and serially connected reduced generalized Cole elements represent a valuable basis for further development of mathematical models for bio-systems and/or any kind of material using the fractional calculus approaches. We propose that this type of powerful modeling tools shall be further applied for noninvasive analysis of complexity of bio-systems and/or any kind of material.
